# Correlation Between Retrograde Trans-Synaptic Degeneration of Ganglion Cells and Optical Coherence Tomography Angiography Following Ischemic Stroke

**DOI:** 10.7759/cureus.19788

**Published:** 2021-11-21

**Authors:** Mercedes Molero-Senosiain, Beatriz Vidal-Villegas, Javier Pascual-Prieto, Clara Valor-Suarez, Federico Saenz-Frances, Enrique Santos-Bueso

**Affiliations:** 1 Ophthalmology, Leicester Royal Infirmary, Leicester, GBR; 2 Ophthalmology, Hospital Clínico San Carlos, Madrid, ESP; 3 Ophthalmology, Complejo Asistencial de Ávila, Ávila, ESP; 4 Ophthalmology, St. Thomas' Hospital, London, GBR

**Keywords:** visual field defect, macular microvascularization, ganglion cells, oct angiography, stroke

## Abstract

Objective

Following nerve injury, the projection of posterior visual pathway lesions into the macular ganglion cell layer (GCL) region indicates retrograde trans-synaptic degeneration (RTSD) as a mechanism of functional damage.

Our purpose is to assess GCL damage and the impacts of ischemic brain lesions affecting the visual pathway on macular microvascularization in patients with stroke.

Methods

In a case-control study, we examined 15 ischemic stroke patients who showed visual field defects and 50 healthy controls using the high-resolution optical coherence tomography (OCT) techniques such as spectral domain-OCT (SD-OCT) to measure retinal nerve fiber layer (RNFL) and GCL thicknesses, and OCT angiography (OCTA) to assess damage to the macular microvasculature.

Results

In the cases, the correlation was detected among the site of vascular damage, visual field defect, retinal GCL thinning, and normal RNFL thickness. Further observations were significant reductions in macular thickness, GCL thickness, outer retinal layer vascular density, and vascular area in deeper retinal layers (p < 0.05).

Conclusion

Our findings suggest that ocular microvasculature abnormalities could serve as diagnostic and/or prognostic markers in patients with stroke and support the described use of GCL thickness as an image marker of visual pathway RTSD after brain injury.

## Introduction

Trans-synaptic degeneration may be seen in post-synaptic neurons (anterograde) and pre-synaptic neurons (retrograde). In the eye, retrograde trans-synaptic degeneration takes place in the retinal ganglion cells that project to the lateral geniculate nucleus after the death of the neurons that synapse with these cells [1].

Stroke is a significant cause of disability and death worldwide. According to the Spanish Society of Neurology (SEN), stroke is the second cause of death in Spain (the first in women), the first cause of acquired disability in adults, and the second cause of dementia. Estimates provided by this society indicate that every year 110,000-120,000 people will experience stroke, and that 50% of these individuals will die or be left with disabling sequelae [2].

According to the guidelines of the different European organisations, multiple classifications of stroke exist depending on criteria such as ischemic/hemorrhagic, brain site, and/or similar cause [3-7].

Ischemic strokes, whereby reduced blood flow causes injury to a given brain region, are a consequence in most cases of a systemic disorder involving both macrovascular and microvascular abnormalities. The main risk factor for both ischemic and hemorrhagic stroke is high blood pressure, followed by dyslipidemia and diabetes mellitus [8,9].

Fundoscopy allows for the direct visualization of retinal microvascular changes. In chronic hypertensive patients, fundoscopy gives clinicians an idea of the extent of systemic disease without the need for invasive tests. Classically, the morphological features of hypertensive retinopathy are stratified according to the classification guidelines of the International Council of Ophthalmology (ICO). There are some limitations to this classification system, however, such as structural changes prior to established clinical signs or interobserver differences.

Since its introduction, spectral domain-optical coherence tomography (SD-OCT) has acquired an important role in the assessment of neuro-ophthalmological diseases. Using this imaging technique, it is possible to assess the integrity of neuronal and cellular axons or their diminished presence related to the progression of some diseases of the visual pathway, as well as to predict visual recovery after surgery in compressive optic neuropathies. SD-OCT imaging is also an important marker of neurodegeneration and is emerging as a promising objective tool for multiple sclerosis trials [1,10].

OCT angiography (OCTA) is a new way to examine the microvasculature of the choroid and retina, otherwise known as broad-spectrum amplitude-decorrelation angiography. This technique detects the diffractive movement of RBCs as changes in the decorrelation signal to create a three-dimensional map of vascularization at different levels (retina, superficial choroid plexus, and deep choroid plexus) that allows a quantitative assessment of the microcirculation. The procedure is non-invasive and does not require pharmacological mydriasis or IV contrast injection.

The main advantages of SD-OCT and the newer technique OCTA over fundoscopy lie in their ability to detect possible changes in microvascularization prior to any clinical signs visible in the fundus exam. A further benefit is that OCT provides numerical values ​​that nullify interobserver differences.

Several studies have shown that in adults due to ageing, there is a decrease in the vascularization of the different capillary plexuses and an increased avascular area of ​​the fovea. Both these signs can be detected by OCTA. In patients with a history of stroke and visual field defects, SD-OCT has been able to correlate this condition with a reduction or loss of ganglion cells. Prospective studies are needed to examine when retinal microvascularization changes take place in these patients [1,10-13].

The present study was designed to assess retrograde trans-synaptic neuron degeneration (RTSD) in ganglion cells by OCTA in patients who have suffered a stroke, causing perimetry changes. Our working hypothesis was that the area of vascularization of the retina and ganglion cell degeneration would differ between stroke patients and control subjects. As secondary objectives, we determined whether a correlation exists between retinal nerve fiber layer (RNFL) thickness and stroke, and confirmed the known relationship between ganglion cell layer (GCL) thickness and visual field defect.

## Materials and methods

For this case-control study, we recruited 15 patients (15 eyes) who had suffered an ischemic stroke and 50 healthy individuals (50 eyes) with no cardiovascular risk factors or other systemic diseases. Both patients and controls gave their written informed consent to participate in the study, whose protocol fulfilled the principles of the Declaration of Helsinki.

Cases were subjects aged over 40 years, who had been diagnosed with a stroke of any aetiology and location in the previous two years, referred to the neuro-ophthalmology unit of the Hospital Clínico San Carlos, Madrid, Spain, in whom we detected a visual field abnormality using a Humphrey 24.2 (Visual Field Analyzer, Carl Zeiss Meditec, Dublin, CA) or Octopus TOP 123 (Haag-Streit Diagnostics, Koenig, Switzerland) perimetry device.

Patients were excluded if they had an ophthalmologic condition that could interfere with the results of the study including established glaucoma, macular disease (macular degeneration, epiretinal membranes or other vascular condition), severe amblyopia, or any condition involving media opacity that could interfere with image quality. Patients were also excluded if they had motor and/or cognitive sequelae determining their poor collaboration. The cases finally selected were 15 individuals with a history of cerebrovascular stroke who were under follow up at the neuro-ophthalmology unit and/or neurology department over the year 2018-2019 fulfilling the inclusion and exclusion criteria.

Cases and controls were subjected to the following tests only once: best-corrected visual acuity (BCVA), intraocular pressure (IOP) by applanation tonometry (Perkins; Clement-Clarke, Haag-Streit, UK), visual field using a Humphrey 24.2 or Octopus TOP, and SD-OCT (Cirrus®, Zeiss, Oberkochen, Germany), and OCTA (RS-3000, Nidek Co., Gamagori, Japan).

OCTA images of the optic nerve head and macular area were obtained by a single trained examiner. In all participants, scans were conducted in one eye. The macular area scanned was 4.5 mm × 4.5 mm at a scan density of 256 A-scans (horizontal) × 256 B-scans (vertical).

**Figure 1:**
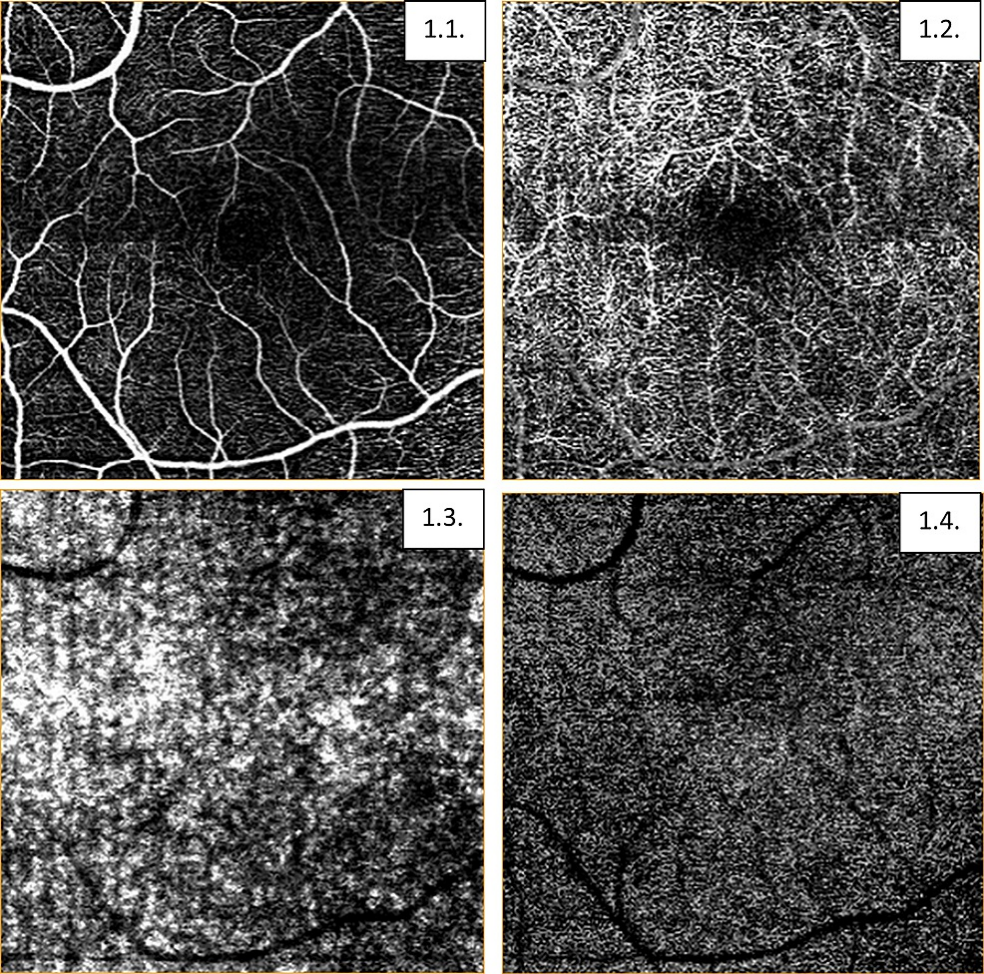
Representative example of macular vascular density analysis with Nidek 3000 in a patient with stroke. 1.1: Radial peripapillary capillary plexus (RPCP); Superficial capillary plexus (SCP); Inner retinal plexus (IRP). 1.2: Deep capillary plexus (DCP). 1.3: Choroid. 1.4: Outer retina.

Macular thickness was analyzed in a 4.5 × 4.5 mm2 field divided into three concentric “rings”, central, parafoveal, and perifoveal, each centred at the fovea of diameters 1.0, 3.0, and 4.5 mm, respectively. The two outer rings were further divided into four quadrants by two orthogonal lines to define nine subfields in total (Figure 1) [14,15].

Statistical analysis

All statistical tests were performed using Windows SPSS 25.0 software (SPSS Inc., Chicago, IL). The Kolmogorov-Smirnov test was used to check the normality of the distribution of quantitative variables, which are expressed as the mean and SD. To compare the demographical characteristics of the case and control groups, we used the Student's t and Chi-squared tests. Data for macular and GCL thickness, vessel density (VD), and vascular area (VA) in each of the layers examined at the level of the macula were compared using the Mann-Whitney U test for independent samples in non-parametric tests. Significance was set at p < 0.05.

## Results

Cases and controls did not differ significantly in terms of mean age which was 59.7 ± 9.02 years in the stroke group and 68.36 ± 9.94 years in the control group (p = 0.297), IOP (p = 0.512) or BCVA (p = 0.067). Proportions of men differed significantly: 34% in the cases and 85% in the controls (p = 0.001) (Table 1).

**Table 1:** Demographic and clinical characteristics of the study cases. Demographic and clinical characteristics of the study cases. BCVA: Best corrected visual acuity; IOP: Intraocular pressure. * Student's t test; + Chi-squared test. Signification p < 0.05

Variable	Strokes n = 15	Controls n = 50	Signification
Sex cases (% men)	34	85	p = 0.001+
Age (mean + SD) (years)	59,7±9,02	65,36±9,94	p = 0.297*
BCVA (mean + SD) (Snellen)	0,56 ± 0,23	0,69 ± 0,21	p = 0.067*
IOP (mean + SD) (mmHg)	15,20 ± 3,03	15,60 ± 1,81	p = 0.512*

In the stroke patients, probable disease etiologies were hypertension recorded in 60%, followed by cardioembolic in 15%, and undetermined cause in 25%. The main site affected was the right posterior cerebral artery (PCA) in 60%, left PCA in 25%; total anterior circulation infarct (TACI) was detected in 15%. Stoke duration measured as the time elapsed since infarction was 17.8 ± 4.97 (14-25 months). The main visual field defect was homonymous hemianopia (80%) followed by homonymous quadrantanopia (20%). A total of 30% of the patients had other sequelae, of which we should mention dysarthria in one and hemiplegia in two.

The macular OCTA map indicated significantly reduced (p < 0.05) outer retinal layer thicknesses in all quadrants and upper and lower inner retina in the cases compared to the controls. GCL thickness was also diminished in the outer (p < 0.05) but not inner retina (Table 2).

**Table 2:** Macular analysis in healthy controls and patients with stroke using Nidek 3000. TS: Temporal superior; NS: Nasal superior; NI: Nasal inferior; TI: Temporal inferior; TS: Temporal superior; NS: Nasal superior. Mann–Whitney U test. Signification p < 0.05.

Variables		Controls n = 50	Cases n = 15	Signification
Macular thickness (microns)	Central	272,82±19,77	277±24,36	p = 0.858
	Inner temporal	329,03±12,01	316,04±23,78	p = 0.175
	Inner superior	324,93±14,23	329,97±12,80	p = 0.006
	Inner nasal	344,82±13,28	327,92±25,98	p = 0.037
	Inner inferior	340,01±12,30	326,07±14,15	p = 0.003
	Outer temporal	298,92±13,98	283,41±14,34	p < 0.001
	Outer superior	313,58±12,51	293,70±12,81	p < 0.001
	Outer nasal	322,20±13,26	301,66±17,15	p < 0.001
	Outer inferior	301,18±15,07	283,45±13,11	p < 0.001
Ganglion cell layer thickness (microns)	Inner TS	69,72±13,72	68,45±22,97	p = 0.537
	Inner NS	74,19±14,93	69,65±15,57	p = 0.372
	Inner NI	79,64±14,42	69,34±18,49	p = 0.60
	Inner TI	75,49±14,56	71,13±25,12	p = 0.929
	Outer TS	114,78±8,31	96,12±20,07	p < 0.001
	Outer NS	122,07±8,43	103,84±17,95	p < 0.001
	Outer NI	112,43±7,72	101,85±20,25	p < 0.001
	Outer TI	116,79±6,77	96,80±24,84	p = 0.002

In patients with stroke, VD was also significantly reduced (p < 0.05) at the level of all vascular plexuses (deep, outer retinal, and choroid), as was the vascular area of the radial and deep peripapillary plexuses (p < 0.05) (Table 3).

**Table 3:** Macular A-OCT Nidek 3000 results in cases with stroke and healthy subjects. RPCP: Radial peripapillary capillary plexus; SCP: Superficial capillary plexus; IPR: Inner retina plexus; DCP: Deep capillary plexus; mm2: squared millimetres. Mann–Whitney U test. Signification p < 0.05.

		Controls (n = 50)	Cases (n = 15)	Signification
Vascular density (%)	RPCP+SCP+IPR	6,21±2,57	4,11±2,06	p = 0.01
	DCP	26,75±5,82	20,08±5,64	p < 0.001
	External retina	42,68±7,97	32,67±7,73	p < 0.001
	Choroid	30,14±12,74	21,93±7,47	p = 0.007
Vascular area (mm^2^)	RPCP+SCP+IPR	3,23±0.47	2,43±0.46	p < 0.001
	DCP	7,17±0.60	6,50±0,53	p < 0.001
	External retina	8,07±0,84	7,86±0,74	p = 0.417
	Choroid	6,86±1,80	7,10±0,52	p = 0.326

## Discussion

In all the patients included in this case-control study, visual field defects were correlated with GCL thinning. This was observed at least one year after the stroke, as reported in other studies [11,12]. Other authors have reported macular or GCL atrophy in patients who have experienced RTSD of visual pathway neurons in the absence of another neurological or ophthalmological condition [11,13]. ​​According to Keller J et al and Jindahra P et al., macular changes seem to occur within the first year of a stroke and remain stable thereafter [13,16-18].

Our RNFL thickness values showed a lower correlation with visual field defect in our patients than with GCL thickness. The reason for this could be that neuron somas in the macula are more numerous and are topographically organized to coincide with the visual field. In contrast, the distribution of fibres of the RNFL is more complex and it seems more difficult to correlate RNFL thickness with stroke than with ocular diseases such as glaucoma [13,16,18]. Inconsistent with our findings, significant RNFL thickness differences have been described in healthy subjects and patients with ischemic brain injury 100.48±13.32 µm versus 75.41 ± 14.53 µm, respectively (p < 0.001) [1].

Following optic nerve damage due to glaucoma via possible anterograde degeneration, there is a clear correlation between RNFL thinning and perimetric defect. However, the role of GCL in anterograde trans-synaptic degeneration is not as clear, although some authors argue that GCL changes can be used for the early diagnosis and/or follow up of glaucoma [19].

Neurological damage caused by other conditions, such as demyelinating or compressive chiasmal tumours, also leads to acquired RTSD [20-28].

In multiple sclerosis, optic nerve damage can be seen in 25-50% of patients with optic neuritis and some axonal degeneration [29,30]. A moderate relationship has been also identified between RNFL loss in patients whose MRI showed some degree of cerebral atrophy. In these patients, generalized GCL thinning has been observed in multiple studies, especially in patients with a history of optic neuritis. Some authors describe GCL measurement as a more sensitive marker than RNFL to detect nerve damage even in the absence of a previous optic neuritis episode [31,32]. This loss of ganglion cells has been also correlated with compromised visual quality, contrast sensitivity, and even quality of life assessed by several questionnaires [20-23].

In other neurodegenerative diseases, such as Alzheimer's or Parkinson's, overall GCL thinning has also been observed. In the case of Parkinson's disease, mainly RNFL thickness and macular thickness are reduced, especially affecting the inner layers [25,26].

In an OCTA study in patients with arterial hypertension, Pascual-Prieto J et al. detected a decrease in macular thickness and VD compared to controls. These authors proposed that these findings of generalized atrophy could be due to chronic ischemia caused by hypertension [14].

Recently, Jaumandreu L et al. reported reduced peripapillary and macular VD concomitant with reduced homonymous GCL thickness and visual field defects in patients with lesions in the retrogeniculate visual pathway [33].

Our study has several limitations. The main limitation is the small number of cases and the fact that these patients had suffered brain injury long before OCTA and showed heterogeneous clinical presentations. A limitation of OCTA is that it only measures blood flow contrast or RBC movements. Nevertheless, to the best of our knowledge, this is the first study to correlate stroke with OCTA images. In future prospective studies, it would be interesting to assess functional and morphological changes in the macular area and optic nerve head produced by stroke affecting the posterior visual pathway. 

Macular imaging by OCT may serve as a marker to quantify damage to the posterior visual pathway. This may be especially useful in patients who are unable to cooperate with functional tests [13,27].

## Conclusions

In conclusion, the mapping of posterior visual pathway lesions and their projection into the macular GCL subfield served to confirm RTSD after brain injury as a mechanism of functional damage. Our findings support the use of GCL thickness as a biomarker of such lesions, as described in the literature. The differences detected here between patients with stroke and healthy subjects in vascular area and vessel density in most layers of the retina provide a new direction for further research.

## References

[1] Park HY, Park YG, Cho AH, Park CK (2013). Transneuronal retrograde degeneration of the retinal ganglion cells in patients with cerebral infarction. Ophthalmology.

[2] Murray CJ, Lopez AD (1997). Mortality by cause for eight regions of the world: global burden of disease study. Lancet.

[3] Rojas JI, Zurru MC, Patrucco L, Romano M, Riccio PM, Cristiano E (2006). [Ischemic stroke registry]. Medicina (B Aires).

[4] Caplan LR (2009). Basic pathology, anatomy, and pathophysiology of stroke. In: Caplan's Stroke: A Clinical Approach. https://www.elsevier.com/books/T/A/9781416047216.

[5] Bogousslavsky J, Van Melle G, Regli F (1988). The Lausanne Stroke Registry: analysis of 1,000 consecutive patients with first stroke. Stroke.

[6] Bamford J, Sandercock P, Dennis M, Warlow C, Burn J (1991). Classification and natural history of clinically identifiable subtypes of cerebral infarction. The Lancet.

[7] Díez-Tejedor E, del Brutto O, Alvarez Sabín J, Muñoz M, Abiusi G (2001). [Classification of the cerebrovascular diseases. Iberoamerican Cerebrovascular diseases Society]. Rev Neurol.

[8] Moreno VP, García-Raso A, García-Bueno MJ, Sánchez-Sánchez C, Meseguer E, Mata R, Llamas P (2008). [Vascular risk factors in patients with ischaemic stroke. Distribution according to age, sex and stroke subtype]. Rev Neurol.

[9] Brea A, Laclaustra M, Martorell E, Pedragosa A (2013). [Epidemiology of cerebrovascular disease in Spain]. Clin Investig Arterioscler.

[10] Chan NC, Chan CK (2018). The role of optical coherence tomography in the acute management of neuro-ophthalmic diseases. Asia Pac J Ophthalmol (Phila).

[11] Hood DC, Fortune B, Arthur SN, Xing D, Salant JA, Ritch R, Liebmann JM (2008). Blood vessel contributions to retinal nerve fiber layer thickness profiles measured with optical coherence tomography. J Glaucoma.

[12] McAuley DL, Russell RW (1979). Correlation of CAT scan and visual field defects in vascular lesions of the posterior visual pathways. J Neurol Neurosurg Psychiatry.

[13] Keller J, Sánchez-Dalmau BF, Villoslada P (2014). Lesions in the posterior visual pathway promote trans-synaptic degeneration of retinal ganglion cells. PLoS One.

[14] Pascual-Prieto J, Burgos-Blasco B, Ávila Sánchez-Torija M (2020). Utility of optical coherence tomography angiography in detecting vascular retinal damage caused by arterial hypertension. Eur J Ophthalmol.

[15] (2018). OCT-Angiography option for NIDEK OCT series. https://www.nidek-intl.com/product/ophthaloptom/diagnostic/dia_retina/angioscan.html.

[16] Jindahra P, Petrie A, Plant GT (2009). Retrograde trans-synaptic retinal ganglion cell loss identified by optical coherence tomography. Brain.

[17] Jindahra P, Petrie A, Plant GT (2012). The time course of retrograde trans-synaptic degeneration following occipital lobe damage in humans. Brain.

[18] Jindahra P, Petrie A, Plant GT (2012). Thinning of the retinal nerve fibre layer in homonymous quadrantanopia: further evidence for retrograde trans-synaptic degeneration in the human visual system. Neuro-ophthalmology.

[19] Michelessi M, Riva I, Martini E (2019). Macular versus nerve fibre layer versus optic nerve head imaging for diagnosing glaucoma at different stages of the disease: Multicenter Italian Glaucoma Imaging Study. Acta Ophthalmol.

[20] Balk LJ, Twisk JW, Steenwijk MD (2014). A dam for retrograde axonal degeneration in multiple sclerosis?. J Neurol Neurosurg Psychiatry.

[21] Gabilondo I, Martínez-Lapiscina EH, Martínez-Heras E (2014). Trans-synaptic axonal degeneration in the visual pathway in multiple sclerosis. Ann Neurol.

[22] Garcia-Martin E, Polo V, Larrosa JM (2014). Retinal layer segmentation in patients with multiple sclerosis using spectral domain optical coherence tomography. Ophthalmology.

[23] Walter SD, Ishikawa H, Galetta KM (2012). Ganglion cell loss in relation to visual disability in multiple sclerosis. Ophthalmology.

[24] Saidha S, Syc SB, Durbin MK (2011). Visual dysfunction in multiple sclerosis correlates better with optical coherence tomography derived estimates of macular ganglion cell layer thickness than peripapillary retinal nerve fiber layer thickness. Mult Scler.

[25] Ohkubo S, Higashide T, Takeda H, Murotani E, Hayashi Y, Sugiyama K (2012). Relationship between macular ganglion cell complex parameters and visual field parameters after tumor resection in chiasmal compression. Jpn J Ophthalmol.

[26] Choi J, Kwon J, Shin JW, Lee J, Lee S, Kook MS (2017). Quantitative optical coherence tomography angiography of macular vascular structure and foveal avascular zone in glaucoma. PLoS One.

[27] He XF, Liu YT, Peng C, Zhang F, Zhuang S, Zhang JS (2012). Optical coherence tomography assessed retinal nerve fiber layer thickness in patients with Alzheimer's disease: a meta-analysis. Int J Ophthalmol.

[28] Mekhasingharak N, Laowanapiban P, Siritho S, Satukijchai C, Prayoonwiwat N, Jitprapaikulsan J, Chirapapaisan N (2018). Optical coherence tomography in central nervous system demyelinating diseases related optic neuritis. Int J Ophthalmol.

[29] Rebolleda G, Diez-Alvarez L, Casado A, Sánchez-Sánchez C, de Dompablo E, González-López JJ, Muñoz-Negrete FJ (2015). OCT: New perspectives in neuro-ophthalmology. Saudi J Ophthalmol.

[30] Kardon RH (2011). Role of the macular optical coherence tomography scan in neuro-ophthalmology. J Neuroophthalmol.

[31] Noval S, Contreras I, Muñoz S, Oreja-Guevara C, Manzano B, Rebolleda G (2011). Optical coherence tomography in multiple sclerosis and neuromyelitis optica: an update. Mult Scler Int.

[32] Scheel M, Finke C, Oberwahrenbrock T (2014). Retinal nerve fibre layer thickness correlates with brain white matter damage in multiple sclerosis: a combined optical coherence tomography and diffusion tensor imaging study. Mult Scler.

[33] Jaumandreu L, Sánchez-Gutiérrez V, Muñoz-Negrete FJ, de Juan V, Rebolleda G (2019). Reduced peripapillary and macular vessel density in unilateral postgeniculate lesions with retrograde transsynaptic degeneration. J Neuroophthalmol.

